# Evaluation of Efficiency of Polymerization, Surface Roughness, Porosity and Adaptation of Flowable and Sculptable Bulk Fill Composite Resins

**DOI:** 10.3390/molecules26175202

**Published:** 2021-08-27

**Authors:** Elizabeta Gjorgievska, Daniel S. Oh, Daewon Haam, Dragana Gabric, Nichola J. Coleman

**Affiliations:** 1 Department of Paediatric and Preventive Dentistry, Faculty of Dental Medicine, University “Ss Cyril and Methodius”, 1000 Skopje, North Macedonia; egjorgievska@stomfak.ukim.edu.mk; 2 College of Dental Medicine, Columbia University, New York, NY 10032, USA; danielsunho@gmail.com (D.S.O.); daewonhaam@gmail.com (D.H.); 3 Department of Oral Surgery, School of Dental Medicine, University of Zagreb, Clinical Hospital Center, 10000 Zagreb, Croatia; 4 Faculty of Engineering and Science, University of Greenwich, Kent ME4 4TB, UK; n.coleman@greenwich.ac.uk

**Keywords:** composite resin, bulk fill, polymerization, surface roughness, porosity, adaptation

## Abstract

A new category of commercial bulk fill composite resins (CRs) enables the placement of 4-mm-thick layers as an alternative to the traditional time-consuming incremental technique. The purpose of the present study was to compare the efficiency of the polymerization, adaptation and porosity of two high-viscosity ‘sculptable’ bulk fill CRs (Filtek™ Bulk Fill (3M™ ESPE, St. Paul, MN, USA) and Tetric EvoCeram^®^ Bulk Fill (Ivoclar Vivadent AG, Schwan, Liechtenstein)) and two low-viscosity ‘flowable’ bulk fill CRs (SureFil^®^ SDR™ flow (Dentsply Sirona, Charlotte, NC, USA) and Tetric EvoFlow^®^ Bulk Fill (Ivoclar Vivadent AG, Schaan, Liechtenstein)). Cylindrical samples of the bulk fill CRs (4 mm height × 10 mm diameter) were analyzed by Fourier-transform infrared spectroscopy (FTIR) and atomic force microscopy (AFM). Additionally, occlusal cavities were prepared in twelve extracted human molars and restored with the bulk fill CRs (*n* = 3 for each CR). The adaptation and porosity of the bulk fill CRs were evaluated by X-ray microcomputed tomography (µCT) with a 3D morphometric analysis, and the adaptation was also analyzed by scanning electron microscopy (SEM) on longitudinal vestibulo-oral sections of the restored teeth. The AFM analysis demonstrated that the surface roughness of the SureFil^®^ SDR™ flow was higher than that of the Tetric EvoFlow^®^ Bulk Fill and that the surface roughness of Filtek™ Bulk Fill was higher than that of Tetric EvoCeram^®^ Bulk Fill. µCT and SEM confirmed that the flowable bulk fill CRs had excellent adaptation to the cavity walls. The 3D morphometric analysis showed the highest and lowest degrees of porosity in Filtek™ Bulk Fill and Tetric EvoFlow^®^ Bulk Fill, respectively. In general, the flowable bulk fill CRs exhibited better adaptation, a higher efficiency of polymerization and lower porosity than the sculptable materials.

## 1. Introduction

The main disadvantages of composite resins (CRs) are their moisture sensitivity and polymerization shrinkage, which lead to the contraction of the total composite volume and the development of polymerization stress [1,2]. The negative effects of polymerization stress (e.g., cusp deflection, marginal gap and dentinal defect formation) can be minimized by using multiple layering techniques and different methods of light polymerization [3]. Since incremental layering and stratifying techniques are time-consuming and technique-sensitive, recently, a new category of ‘bulk fill’ CRs has been developed to enable the clinician to place restorations up to 4 mm thick without negatively affecting the cavity adaptation, shrinkage and efficiency of polymerization [4].

Bulk fill CRs are further classified according to their rheological properties into base or full-body types for the restoration of posterior teeth [5]. According to Hirata et al. [3], there are two clinical approaches for using bulk fill materials to restore posterior teeth. The first is to use a ‘sculptable’ high-viscosity bulk fill restorative in a single increment in cavity preparations up to 4 mm deep, and the second is to apply a ‘flowable’ low-viscosity resin as a base material for dentin replacement in a single increment, which is then finished with a final layer of conventional CR to restore the enamel.

The bulk fill CRs have a similar composition to their conventional counterparts (i.e., a photocurable blend of bifunctional methacrylate monomers and silanized glass fillers) with enhanced translucency arising from lower filler concentrations and larger particle sizes [6,7,8]. In addition to improving the translucency, an increased depth of cure is also achieved by using specific polymerization modulators and/or by using more potent photo-initiator systems [9]. Nevertheless, several studies have demonstrated that the deeper layers do not achieve optimal polymerization due to light attenuation [6,7,10], which is determined by absorption by photo-initiators and pigments, the reflection on filler/resin interfaces, changes in refractive indices during polymerization and temperature changes, as well as air voids incorporated into the structure of the composite material [6].

The presence of porosities (voids) in CRs may arise from the manufacturing process or the application technique (i.e., condensing of the material into the cavity) [11,12] and could result in fractures of the restoration [13], microleakage and increased surface roughness [14]. Voids are commonly found along the junction between the CR layers when the incremental technique is applied [15,16]. Additionally, spherical and well-defined voids are also regularly found in materials extruded from the original syringes [15]. It is acknowledged that the higher the viscosity of the composites, the more difficult it becomes to condense them into the prepared cavity.

Typically, voids in CRs have been assessed by the visualization of 300-µm-thick sections under a stereomicroscope. The principal limitation of this technique is that only twenty-five percent of the surface is visible for analysis, and a mathematical relationship is then required to estimate the total number of voids and porosity in the whole sample [17]. With the recent development of high-resolution microcomputed tomography (µCT) with a 3D morphometric analysis, the whole sample can now be assessed and visualized directly, thereby overcoming the limitations of the previous technique. µCT is a nondestructive X-ray technique that enables an accurate 3D reconstruction of the interior and interfaces of CRs for the visualization and analysis of the marginal adaptation, porosity and volumetric change [17].

Changes in the matrix composition; the filler content; the introduction of new monomer and variations in the particle size, type and morphology can all increase the surface roughness of composites [18] and can result in plaque accumulation, gingival inflammation and surface staining [19]. Hence, the purpose of the present study was to evaluate the efficiency of polymerization, surface roughness, porosity and marginal adaptation of two high-viscosity ‘sculptable’ bulk fill CRs (Filtek™ Bulk Fill (3M™ ESPE, St. Paul, MN, USA) and Tetric EvoCeram^®^ Bulk Fill (Ivoclar Vivadent AG, Schaan, Liechtenstein)) and two low-viscosity ‘flowable’ bulk fill CRs (SureFil^®^ SDR™ flow (Dentsply Sirona, Charlotte, NC, USA) and Tetric EvoFlow^®^ Bulk Fill (Ivoclar Vivadent AG, Schaan, Liechtenstein)).

The efficiency of polymerization of the four commercial bulk fill CRs was determined between the top and bottom layers of cylindrical samples (4 mm height × 10 mm diameter) by Fourier-transform infrared spectroscopy (FTIR), and the surface roughness was analyzed on the same samples by atomic force microscopy (AFM). The adaptation and porosity of the bulk fill CRs were evaluated by a µCT with 3D morphometric analysis on the occlusal restorations preformed on extracted human molars. The adaptation was also analyzed by scanning electron microscopy (SEM) on longitudinal vestibulo-oral sections of the restored teeth.

## 2. Materials and Methods

**Figure 1:**
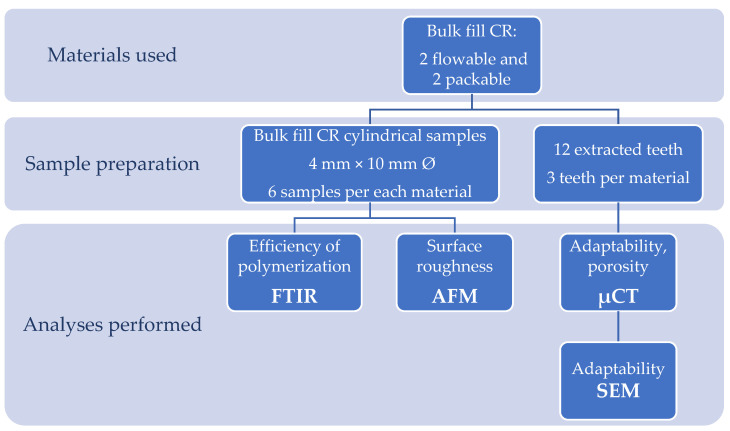
Study design.

This study was carried out in order to analyze four commercial bulk fill CRs (viz., Filtek™ Bulk Fill (3M™ ESPE, St. Paul, MN, USA), Tetric EvoCeram^®^ Bulk Fill (Ivoclar Vivadent AG, Schaan, Liechtenstein), SureFil^®^ SDR™ flow (Dentsply Sirona, Charlotte, NC, USA) and Tetric EvoFlow^®^ Bulk Fill (Ivoclar Vivadent AG, Schaan, Liechtenstein)). It consisted of two parts: first, the analysis of the efficiency of the polymerization by FTIR and the surface roughness by AFM on the cylindrical samples, and second, an analysis of the adaptation and porosity by µCT and SEM on the restorations performed on extracted human teeth (Figure 1).

### 2.1. Fourier-Transform Infrared Spectroscopy (FTIR) & Atomic Force Microscopy (AFM)

The cylindrical samples were prepared from two sculptable CRs (Filtek™ Bulk Fill (3M™ ESPE, St. Paul, MN, USA) and Tetric EvoCeram^®^ Bulk Fill (Ivoclar Vivadent AG, Schaan, Liechtenstein) and two flowable CRs (SureFil^®^ SDR™ flow (Dentsply Sirona, Charlotte, MN, USA) and Tetric EvoFlow^®^ Bulk Fill (Ivoclar Vivadent AG, Schaan, Liechtenstein)).

The materials were placed in silicon molds (4 mm height × 10 mm diameter), covered with celluloid strips on both sides and light-cured on the upper side for 40 s at 1200 mW cm^−2^ (Bluephase20i, Ivoclar Vivadent AG, Schaan Liechtenstein), according to the manufacturer’s instructions. Six samples were prepared of each bulk fill CR. The FTIR and AFM analyses were performed on each sample.

In order to determine the efficiency of polymerization (ISO 4049:2019, 5.2.8., depth-of-cure), FTIR spectra were obtained from the top and bottom layers of each sample using a Perkin Elmer Spectrum Two spectrometer with a Universal Diamond attenuated total reflectance attachment (Waltham, MA, USA) [20,21,22]. The spectra were recorded with 16 accumulated scans between 4000 cm^−1^ and 450 cm^−1^ wavenumbers at a resolution of 4 cm^−1^. The efficiency of the polymerization between the top and the bottom layers was estimated by comparing the ratios of the intensities of the FTIR peaks for the reactive polymerizing C=C bond (at 1635 cm^−1^) and the invariant C=O bond (at 1718 cm^−1^) in the cured polymer and monomer using the following equation [21]:

(1) $$\mathrm{Efficiency}\;\mathrm{ofpolymerization}\;\mathrm{between}\;\mathrm{top}\;\&\;\mathrm{bottom}\;\mathrm{layer}\;\left(\%\right)\;=\frac{\left(\left[C=C\right]\mathrm{top}\;\mathrm{layer}\right)/\left(\left[C=O\right]\mathrm{top}\;\mathrm{layer}\right)}{\left(\left[C=C\right]\mathrm{bottom}\;\mathrm{layer}\right)/\left(\left[C=O\right]\mathrm{bottom}\;\mathrm{layer}\right)}\;\times 100$$

AFM was performed using a Nanosurf Easyscan 2 FlexAFM instrument (Nanosurf AG, Liestal, Switzerland) in tapping mode with a silicon tip at an ambient temperature. The scans were carried out at a pixel resolution of 512 × 512 to generate a topographic map of the surface features.

### 2.2. Scanning Electron Microscopy (SEM) and Microcomputed Tomography (µCT)

Additionally, the four commercial bulk fill CRs were used to restore twelve extracted human permanent molars (*n* = 3 for each bulk fill CR) (Table 1) to enable an evaluation of their adaptation and porosity by µCT and SEM. This study was approved by the Ethic Committee, Faculty of Dental Medicine, University “Ss Cyril and Methodius”, RNM, approval code 09/1335.

The teeth were cleaned by removing the soft tissue and hard deposits attached to the surface and then stored in 0.1% thymol solution. The teeth samples were prepared as follows: the roots were cut with a diamond bur (Diamond Tapered 859 L Medium/5, 314 014, Hager & Meisinger, Neuss, Germany) with a high-speed dental handpiece at the cemento-enamel junction, and the remnants of the pulp tissue were discarded. The coronal segments were thoroughly ultrasonicated (KaVo SONICflex 2003 L, KaVo Dental SAS, Biberach/Riss, Germany) and polished with One-step PoGo (Dentsply Sirona, Charlotte, NC, USA). The cavities were prepared on the occlusal surface of each tooth using a regular grit round diamond bur (No. 801, ISO 806 204 001 514 023, Hager & Meisinger, Neuss, Germany) and a high-speed dental handpiece, followed by a slow-speed handpiece and large-sized round stainless-steel bur (No. 1RF, ISO 310 204 001 001 023, Hager & Meisinger, Neuss, Germany), according to the conventional dental techniques. The cavities were prepared depending on the morphology of the extracted molars with caution to prevent pulp communication at the bottom of the cavity. Then, the teeth were randomly divided into 4 groups, self-etch adhesive was applied and restorations were performed, as listed in Table 1. The sculptable bulk fill CRs were placed in a single layer, while the flowable materials were capped by a layer of a sculptable CR as an enamel substitute. The materials were light-cured for 40 s at 1200 mW cm^−2^ (Bluephase20i, Ivoclar Vivadent AG, Schaan, Liechtenstein), and the final restorations were polished with One-step PoGo Dentsply Sirona, Charlotte, NC, USA).

The instrument used for µCT imaging was a SkyScan 1272 X-ray microscope (Bruker, Kontich, Belgium), and the 3D morphometric analysis was performed on dedicated software (Data Viewer, CTAN and CTVOL software version 1.1.3, Bruker, Kontich, Belgium) to evaluate the porosity and marginal adaptation of the bulk fill CRs.

Each tooth sample was positioned in a specimen holder and fixed by wax with the purpose of avoiding sample movement during the experiment. The operating parameters used for µCT imaging were as follows: source voltage = 100 kV, source current = 100 µA, image pixel size = 6 µm, depth = 16 bit, exposure = 1360 ms and Cu filter = 0.11 mm. For the evaluation of the total porosity, the ‘volume of interest’ was selected as the bulk fill restorative only.

Following a nondestructive µCT analysis, the teeth were then cut in half along the longitudinal axis in a vestibulo-oral direction. The sectioned surfaces were polished with water-cooled carborundum discs (320, 600 and 1200 grit alumina papers, Buehler, Uzwil, Switzerland) and polished with diamond polishing paper (3M™ Polishing Paper 1 Micron 8000 Grit, 3M™ ESPE, St. Paul, MN, USA), and each sample was imaged uncoated using a cold cathode field-emission gun scanning electron microscope (FEG-SEM Hitachi SU 8030, Hitachi, Tokyo, Japan). The samples were previously pumped down in a vacuum desiccator until a sufficient vacuum was achieved to obtain an image by FEG-SEM using secondary electrons (SE) at magnifications up to 2000×.

### 2.3. Statistical Analysis

The statistical analysis was performed with one-way ANOVA followed by Tukey’s post hoc HSD test. The level of significance was set at *p* > 0.05.

## 3. Results

The efficiency of polymerization between the top and the bottom layers (i.e., at a depth of 4 mm) for each bulk fill CR was estimated by comparing the ratios of the intensities of the FTIR peaks for the reactive polymerizing C=C bond (at 1635 cm^−1^) and the invariant C=O bond (at 1718 cm^−1^) in the cured polymer and monomer (Table 2). The lowest value of 78.07 ± 1.46% was recorded for Tetric EvoCeram^®^ Bulk Fill (Ivoclar Vivadent AG, Schaan, Liechtenstein), although this was not statistically different from that of the other sculptable CR at 80.87% ± 2.05%, Filtek™ Bulk Fill (3M™ ESPE, St. Paul, MN, USA). The results showed a significantly higher efficiency of polymerization in the flowable bulk fill CRs in comparison with that of the sculptable materials, with Tetric EvoFlow^®^ Bulk Fill (Ivoclar Vivadent AG, Schaan, Liechtenstein) demonstrating the most effective degree of polymerization at a depth of 4 mm (94.50% ± 0.82%).

**Figure 2:**
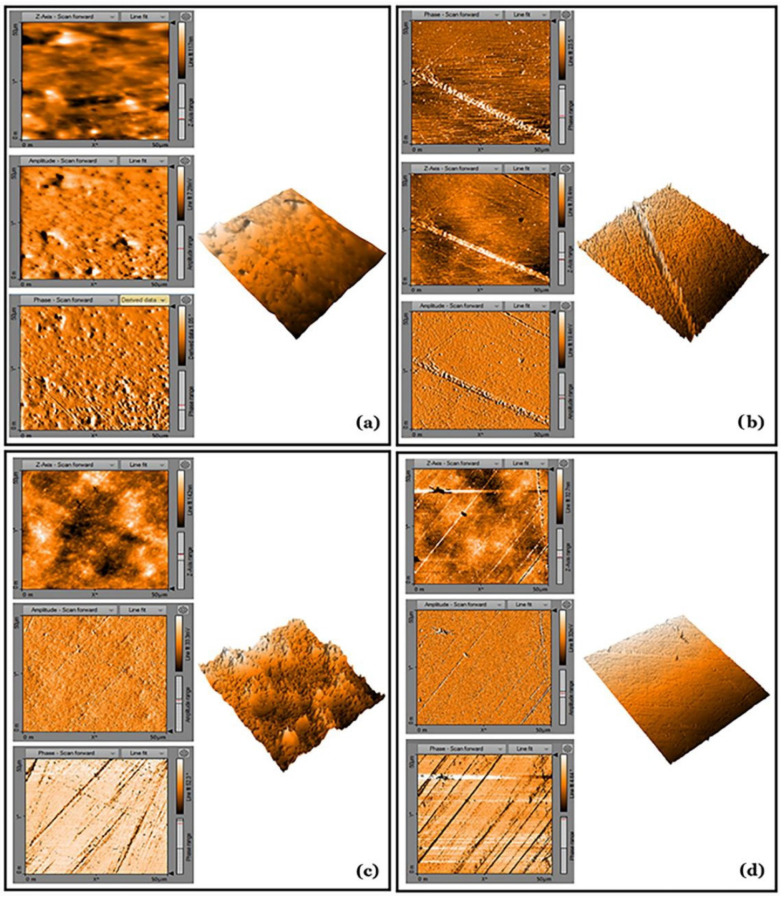
AFM photographs of the surface topography of the bulk fill CR samples: (**a**) Filtek™ Bulk Fill (3M™ ESPE, St. Paul, MN, USA, (**b**) Tetric EvoCeram^®^ Bulk Fill (Ivoclar Vivadent AG, Schaan, Liechtenstein), (**c**) SureFil^®^ SDR^®^ flow (Dentsply Sirona, Charlotte, NC, USA) and (**d**) Tetric EvoFlow^®^ Bulk Fill (Ivoclar Vivadent AG, Schaan, Liechtenstein).

The AFM analysis (Figure 2) demonstrated that the surface roughness of SureFil^®^ SDR™ flow (Dentsply Sirona, Charlotte, NC, USA) was higher than that of Tetric EvoFlow^®^ Bulk Fill (Ivoclar Vivadent AG, Schaan, Liechtenstein) and that the surface roughness of Filtek™ Bulk Fill (3M™ ESPE, St. Paul, MN, USA) was higher than that of Tetric EvoCeram^®^ Bulk Fill (Ivoclar Vivadent AG, Schaan, Liechtenstein).

**Figure 3:**
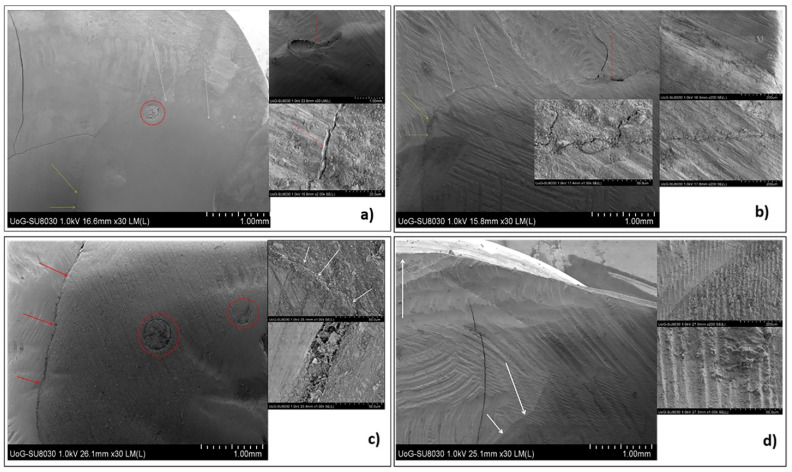
SEM microphotographs of the longitudinal sections of the restored teeth (white arrows are areas of good adaptation, yellow arrows are areas of moderate adaptation, red arrows are areas of inadequate adaptation and red circles are the presence of voids within the material): (**a**) Adper Easy Bond™+Filtek™ Bulk Fill (3M™ ESPE, St. Paul, MN, USA), (**b**) AdheSE^®^+Tetric EvoCeram^®^ Bulk Fill (Ivoclar Vivadent AG, Schaan, Liechtenstein), (**c**) Prime&Bond NT^®^+SureFil^®^ SDR^®^ flow+TPH Spectra ST^®^ (Dentsply Sirona, Charlotte, NC, USA) and (**d**) AdheSE^®^+Tetric EvoFlow^®^ Bulk Fill+Tetric EvoCeram^®^ Bulk Fill (Ivoclar Vivadent AG, Schaan, Liechtenstein).

**Figure 4:**
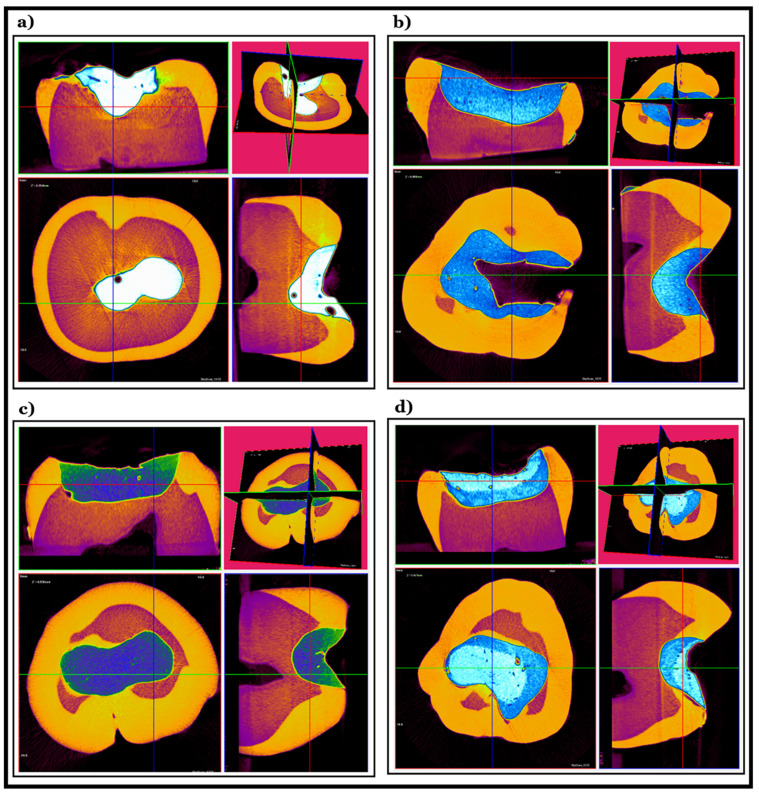
µCT photographs of the restored teeth: (**a**) Adper Easy Bond™+Filtek™ Bulk Fill (3M™ ESPE, St. Paul, MN, USA)—the presence of large air voids within the material, (**b**) AdheSE^®^+Tetric EvoCeram^®^ Bulk Fill (Ivoclar Vivadent AG, Schaan, Liechtenstein)—the presence of small air voids dispersed within the entire restorative material, (**c**) Prime&Bond NT^®^+SureFil^®^ SDR^®^ flow+TPH Spectra ST^®^ (Dentsply Sirona, Charlotte, NC, USA)—a defect in the adaptation towards the cavity wall at the bottom of the cavity, with air voids pronounced at the interface between the flowable and sculptable CR layer and (**d**) AdheSE^®^+Tetric EvoFlow^®^ Bulk Fill+Tetric EvoCeram^®^ Bulk Fill (Ivoclar Vivadent AG, Schaan, Liechtenstein)—an excellent adaptation of the flowable CR towards the cavity walls, with the presence of a large quantity of air voids within the sculptable CR, especially in between layers.

The µCT and SEM microphotographs showed that the flowable bulk fill CRs had excellent adaptation to the cavity walls, particularly compared to that of the sculptable materials (Figure 3 and Figure 4, Videos S1–S4). The best cavity adaptation was observed for Tetric EvoFlow^®^ Bulk Fill (Ivoclar Vivadent AG, Schaan, Liechtenstein).

The highest porosities were observed within the body of the sculptable materials (Figure 2 and Figure 3). Small air voids were also noted at the bottom of the cavities and at the interface between the flowable and sculptable materials (Figure 3 and Figure 4). A quantitative 3D morphometric analysis of the µCT data confirmed that the levels of porosity were significantly higher in the sculptable bulk fill CRs (Table 3). The total porosity was observed to increase in the following order: Tetric EvoFlow^®^ Bulk Fill (Ivoclar Vivadent AG, Schaan Liechtenstein) < SureFil^®^ SDR™ flow (Dentsply Sirona, Charlotte, NC, USA) < Tetric EvoCeram^®^ Bulk Fill (Ivoclar Vivadent AG, Schaan, Liechtenstein) < Filtek™ Bulk Fill (3M™ ESPE, St Paul, MN, USA).

## 4. Discussion

The bulk fill CRs present a recent group of posterior restorative materials that can be directly light-cured to a depth of 4 mm [23]. Due to the possibility of incorporating a greater thickness of CR into a tooth cavity preparation, the bulk fill CRs have become more attractive than their conventional CR counterparts that require incremental placement [3]. The principal advantages of the direct placement and polymerization of the bulk fill CRs are the reduction in the inter-layer voids, bubbles and impurities associated with conventional stratified CR restorations and, also, the associated reduction in clinical time [3].

For a clinician to confidently change from using a traditional incremental filling technique to the bulk filling method, credible clinical trials and laboratory studies comparing the characteristics of the polymerization reaction at restoration depths that simulate the clinical scenario should be performed [24]. In order to assess the increment thickness of bulk fill CRs that could be polymerized efficiently, researchers have referred to ‘depth of cure’ [25] and ‘degree of conversion’ measurements [25]. The methods based on vibrational spectroscopy, such as FTIR (which was used in the current study), are considered more accurate, because they directly quantify the relative proportion of unreacted C=C bonds when the network is crosslinked [26]. The lowest value of efficiency of polymerization in this study was observed for Tetric EvoCeram^®^ Bulk Fill (Ivoclar Vivadent AG, Schaan, Liechtenstein), while higher values were found in the flowable bulk fill CRs compared to the sculptable restoratives. In this respect, Tetric EvoFlow^®^ Bulk Fill (Ivoclar Vivadent AG, Schaan, Liechtenstein) exhibited the highest efficiency of polymerization at 94.50% ± 0.82%. This is in accordance with previous findings that bulk fill CRs were partially likely to fulfil the important requirement regarding being properly cured at a cavity depth of 4 mm (measured by depth of cure and/or degree of conversion). In general, low-viscosity bulk fill CRs performed better regarding polymerization efficiency compared to the high-viscosity bulk fill CRs [27].

However, the simplification of the application technique in bulk fill CRs should not lead to the introduction of inferior materials. As previously mentioned, changes in the matrix composition and filler content; the introduction of new monomers and the variations in particle size, type and morphology can all increase the surface roughness of composites [23], which can cause plaque accumulation, gingival inflammation and surface staining [28]. The AFM analysis showed that the roughness of Tetric EvoFlow^®^ Bulk Fill (Ivoclar Vivadent AG, Schaan, Liechtenstein) was lowest, while SureFil^®^ SDR™ flow (Dentsply Sirona, Charlotte, NC, USA) exhibited the highest surface roughness, probably because it is a flowable bulk fill CR with a complex formulation of urethane dimethacrylate resin, dimethacrylate resin, di-functional diluents, barium- and strontium-alumino-fluoro-silicate glasses (68 wt%, 45 vol%), photo-initiating components and colorant. The possible reason for the high surface roughness could be because of lower filler loading, a greater particle size and polymerization modulators that are chemically embedded in the center of the polymerizable resin that is the backbone of the material [29]. Therefore, on the basis of this high surface roughness, SureFil^®^ SDR™ flow (Dentsply Sirona, Charlotte, NC, USA) should be recommended for use as a dentine substitute in large fillings with a replacement layer of enamel composite.

With respect to the sculptable bulk fill CRs, Tetric EvoCeram^®^ Bulk Fill (Ivoclar Vivadent AG, Schaan, Liechtenstein) was found to have a lower surface roughness compared to that of Filtek™ Bulk Fill (3M™ ESPE, St. Paul, MN, USA). A previous study reported that the roughness of Tetric EvoCeram^®^ Bulk Fill (Ivoclar Vivadent AG, Schaan, Liechtenstein) observed by AFM was virtually indiscernible from that of the surrounding tooth structure [29].

An in vitro study showed that bacteria accumulate in voids [30], and the SEM analysis of three-year-old resin restorations indicated a bacterial collection in the exposed surface pores of the restorations [15]. In addition to the intrinsic properties of the restorative material, surface roughness may also arise from the inadvertent incorporation of voids (porosities) during manufacture or by the clinician during restoration placement [31,32] due to the technique of condensing and smearing the material into the cavity [22].

Stress concentrations in the material enclosing voids can lead to fracture nucleation. When these fractures propagate and connect voids together, this leads to the formation of throughgoing fractures and eventual bulk failure of the rock mass [32]. The presence of voids between incremental layers of the composite material also has an adverse effect on the flexural strength of the restoration [20]. The presented results obtained by µCT show that the porosities were observed predominantly in the sculptable restoratives within the body of the material, at the interface between the flowable and sculptable materials and at the bottom of the cavity. Voids located at the tooth–restoration interface could be mistaken as secondary caries due to the radiolucency of this type of defect [20].

The levels of porosity calculated by the 3D morphometric analysis of the µCT data were significantly higher in the sculptable, rather than in the flowable bulk fill, CRs. The short- and long-term effects of the presence of voids in the materials are varied and depend on the volume, number and location of the voids [16].

## 5. Conclusions

The present study compared the efficiency of polymerization, adaptation and porosity of two high-viscosity ‘sculptable’ bulk fill CRs (Filtek™ Bulk Fill (3M™ ESPE, St. Paul, MN, USA) and Tetric EvoCeram^®^ Bulk Fill (Ivoclar Vivadent AG, Schaan, Liechtenstein)) and two low-viscosity ‘flowable’ bulk fill CRs (SureFil^®^ SDR™ flow (Dentsply Sirona, Charlotte, NC, USA) and Tetric EvoFlow^®^ Bulk Fill (Ivoclar Vivadent AG, Schaan, Liechtenstein)). The following conclusions can be drawn from the findings of this study: (i) both sculptable and flowable bulk fill CRs offer adequate levels of polymerization up to a depth of 4 mm; (ii) with the exception of SureFil^®^ SDR™ flow (Dentsply Sirona, Charlotte, NC, USA), the surface roughness of the bulk fill CRs is low and should protect against bacterial accumulation; (iii) the extent of the porosity is markedly lower in the flowable materials and (iv) the marginal adaptation of the flowable bulk fill restoratives is superior to that of their sculptable counterparts.

## Figures and Tables

**Table 1:** Materials used for the restorations of the extracted human molars.

Consistency of CR	Group	CR	Adhesive
Sculptable	Group 1	Filtek™ Bulk Fill (3M™ ESPE, St. Paul, MN, USA)	Adper™ Easy Bond (3M™ ESPE, St. Paul, MN USA)
	Group 2	Tetric EvoCeram^®^ Bulk Fill (Ivoclar Vivadent AG, Schaan, Liechtenstein)	AdheSE^®^ (Ivoclar Vivadent AG, Schaan, Liechtenstein)
Flowable	Group 3	SureFil^®^ SDR™ flow (Dentsply Sirona, Charlotte, NC, USA) and TPH Spectra^®^ ST flow (Dentsply Sirona, Charlotte, NC, USA)	Prime&Bond^®^ NT (Dentsply Sirona, Charlotte, NC, USA)
	Group 4	Tetric EvoFlow^®^ Bulk Fill (Ivoclar Vivadent AG, Schaan, Liechtenstein) and Tetric EvoCeram^®^ Bulk Fill (Ivoclar Vivadent AG, Schaan, Liechtenstein)	AdheSE^®^ (Ivoclar Vivadent AG, Schaan, Liechtenstein)

**Table 2:** Efficiency of polymerization (derived from the FTIR analysis).

Material		Polymerization Efficiency (%) Mean ± SD
Sculptable	Filtek™ Bulk Fill (3M™ ESPE, St. Paul, MN, USA)	80.87 ± 2.05 ^a^
	Tetric EvoCeram^®^ Bulk Fill (Ivoclar Vivadent AG, Schaan, Liechtenstein)	78.07 ± 1.46 ^bc^
Flowable	SureFil^®^ SDR™ flow (Dentsply Sirona, Charlotte, NC, USA)	86.63 ± 0.93 ^bc^
	Tetric EvoFlow^®^ Bulk Fill (Ivoclar Vivadent AG, Schaan, Liechtenstein)	94.50 ± 0.82 ^ac^

Statistically significant differences (with respect to a one-way ANOVA followed by Tukey’s post hoc honest significant difference test) are denoted by identical superscripts.

**Table 3:** Material porosity (derived from a 3D morphometric analysis of the µCT data).

Material		Material Porosity (%) Mean ± SD
Sculptable	Filtek™ Bulk Fill (3M™ ESPE, St. Paul, MN, USA)	10.92 ± 0.28% ^a^
	Tetric EvoCeram^®^ Bulk Fill (Ivoclar Vivadent AG, Schaan, Liechtenstein)	9.51 ± 0.23% ^a^
Flowable	SureFil^®^ SDR™ flow (Dentsply Sirona, Charlotte, NC, USA)	2.05 ± 0.07% ^a^
	Tetric EvoFlow^®^ Bulk Fill (Ivoclar Vivadent AG, Schaan, Liechtenstein)	0.33 ± 0.08% ^a^

Statistically significant differences (with respect to the one-way ANOVA followed by Tukey’s post hoc honest significant difference test) are denoted by identical superscripts.

## Data Availability

The data are available from the authors.

## Supplementary Materials

The following are available online. Video S1. µCT 3D-rendered model of a restoration performed by Filtek™ Bulk Fill (3M™ ESPE, St. Paul, MN, USA) isolated from the tooth structure. Video S2. µCT 3D-rendered model of a restoration performed by Tetric EvoCeram^®^ Bulk Fill (Ivoclar Vivadent AG, Schaan, Liechtenstein) isolated from the tooth structure. Video S3. µCT 3D-rendered model of a restoration performed by Tetric EvoFlow^®^ Bulk Fill+Tetric EvoCeram^®^ Bulk Fill (Ivoclar Vivadent AG, Schaan, Liechtenstein) isolated from the tooth structure. Video S4. µCT 3D-rendered model of a restoration performed by SureFil^®^ SDR^®^ flow+TPH Spectra ST^®^ (Dentsply Sirona, Charlotte, NC, USA) isolated from the tooth structure.

## Sample Availability

Samples of the compounds are available from the authors.
